# In Vitro Characterization of Canine Microfragmented Adipose Tissue Non-Enzymatically Extracted from the Thigh and Lumbar Regions

**DOI:** 10.3390/ani11113231

**Published:** 2021-11-12

**Authors:** Francesco De Francesco, Valentina Riccio, Reetuparna Biswas, Alice Busato, Caterina Di Bella, Evelina Serri, Andrea Sbarbati, Barbara Zavan, Michele Riccio, Angela Palumbo Piccionello

**Affiliations:** 1 Hand Surgery Unit, Department of Plastic and Reconstructive Surgery, Azienda ‘Ospedali Riuniti di Ancona’, 60126 Ancona, Italy; michele.riccio@ospedaliriuniti.marche.it; 2 School of Biosciences and Veterinary Medicine, University of Camerino, 62032 Matelica, Italy; valy.riccio91@gmail.com (V.R.); caterina.dibella@unicam.it (C.D.B.); evelina.serri@unicam.it (E.S.); angela.palumbo@unicam.it (A.P.P.); 3 Department of Neuroscience, Biomedicine and Movement, Human Anatomy and Histology Section, University of Verona, 37179 Verona, Italy; reetuparna.biswas@univr.it (R.B.); alice.busato@univr.it (A.B.); andrea.sbarbati@univr.it (A.S.); 4 Department of Morphology, Surgery and Experimental Medicine, University of Ferrara, 44121 Ferrara, Italy; barbara.zavan@unife.it

**Keywords:** microfragmented adipose tissue, micrografts, Rigenera technology, adipose stem cells, canine adipose tissue

## Abstract

**Simple Summary:**

Mesenchymal stem cells are located in bone marrow, adipose tissue, synovial membrane, and muscular tissue. They have an immunosuppressive, anti-inflammatory, and antifibrotic effect. Tissue engineering considers the usage of mesenchymal stem cells as a possible option for regenerating tissues, with respect to bone and cartilage, due to their ability to differentiate into multiple cytotypes (including chondrocytes and osteoblasts). Herein, we characterize a non-invasive solution based on Rigenera^®^ technology, a mechanical disaggregation method able to produce autologous adipose tissue-derived micrografts which are analogous to adipose-derived stem cells.

**Abstract:**

Within the adult canine population, disabilities and symptoms including joint pain and functional impairment are commonly observed in articular cartilage lesions and present a challenging feat in the operating room. Clinical settings require less invasive and more minimally manipulated measures facilitated by innovative and advanced technology. Mesenchymal stem cells have recently been proposed and, furthermore, autologous adipose tissue administration via injection has emerged as a new albeit somewhat controversial therapeutic tool. The purpose of this study is to characterize canine autologous micro-fragmented adipose tissue (micrografts) by mechanical approach without substantial manipulations. Adipose tissue samples collected from six dogs were processed by a Rigenera device and by enzymatic digestion from two different body regions (lumbar and thigh region). Interestingly, the immunophenotypic analysis attested that cells from Rigenera^®^ were highly positive for the mesenchymal stem cells markers CD73 and CD90, less positive for hematopoietic CD45 and CD34, and negative for MHC class II antibodies (which play a role in immune responses). Finally, the Rigenera^®^ technology obtained micrografts with a 35% higher expression of the IL10 gene with relevant anti-inflammatory activities compared to the enzymatic digestion protocol. This evidence suggests a potential improved clinical outcome capable of modulating inflammation and immune responses.

## 1. Introduction

Osteoarthrosis (OA) is a common degenerative, chronic, inflammatory, painful, and disabling condition which affects the joints. High rates of OA have been observed in dogs [1,2] and in 20% of the canine population older than one year presenting OA across different stages [3,4]. OA is one of the most disabling diseases in dogs [5,6,7,8,9] and is characterized by lameness, chronic pain, and functional impairment with a reduced quality of life. OA ultimately results in a reduction of mobility and can result in the complete loss of motor function [10]. Treatment of OA aims mainly to reduce pain and inflammation through drug administration, appropriate diets, and physiotherapeutic sessions. Typically, the non-operative approach is the most common. However, when this fails, surgical treatment can be performed [11,12,13,14,15]. Nonetheless, OA is still today the main cause of non-traumatic euthanasia in dogs since pharmacological drugs (FANS/FAS) mitigate articular pain without affecting OA progression. In recent years, scientific research has focused on substances able to slow down the progression of OA, regenerating the damaged tissues through local delivery instead of oral or parenteral drug administration, to avoid systematic side effects. For this purpose, the use of oral visco-supplementation and intra-articular substances such as hyaluronic acid, platelet rich plasma (PRP), and mesenchymal stem cells (MSC) have increased over time [16,17]. MSCs are located in the bone marrow, adipose tissue, synovial membrane and muscular tissue, exerting an immunosuppressive, anti-inflammatory, and antifibrotic effect. Tissue engineering considers adopting MSCs as an option for regenerating tissues, with reference to bone and cartilage, due to their ability to differentiate into multiple cytotypes, including chondrocytes and osteoblasts [18,19], but MSCs are also able to heal the damaged tendon [20]. Therefore, the “stromal vascular fraction” (SVF), which is mainly located around blood vessels, is a heterogeneous solution stemmed from adipose tissue and composed by adipose stem cells (ASCs), endothelial cells, and stromal cells. [21]. According to the literature, ASCs are genetically and morphologically stable in long-term cultures and are characterized by slow senescence and a high proliferation rate [22,23,24]. Cell therapies and “minimally manipulated” tissue micrografts mainly differ in that the adipose tissue is not enzymatically digested but only processed mechanically. Autologous adipose micrografts consequently contain MSCs and an extra-cellular matrix (ECM) [25,26]. Mechanically-obtained micrografts approaches are considered advantageous compared to enzymatic digestion treatments due to the ability to preserve the stromal vascular niche, permitting adequate growth factor release as well as discharging bioactive molecules by exosomes rich in mechanically processed fat. Furthermore, mechanically obtained-Adipose SVF techniques can retain the structure and morphology of the micro-environment where micrografts reside. Previous studies regarding “minimally manipulated” adipose tissue administration have confirmed its safety [27,28], but certain issues are not as clear-cut. The benefits, for instance, of SVF in repairing tissue and its bridging potential of new and old tissue is well-known, but these advantages are not as appropriate concerning intra-articular fat injections due to the lack of tissue fragments to connect [29]. Interestingly, SVF possesses key trophic, anti-apoptotic, anti-scarring, mitogenic and immunomodulatory properties [30,31] which aid in generating numerous bioactive elements as well as growth factors and cytokines. Such cells can perceive and mark modifications within the specific microenvironment [32]. The field of research has also addressed the use of purified adipose tissue and revealed positive anti-inflammatory and reconstructive outcomes related to cartilage regeneration [33] with in vitro and in vivo studies. Good manufacturing practice regulations [34] exert restrictions on enzymatic therapy, and therefore minimally manipulated autologous adipose tissue is a favorable treatment alternative. In this context, micro-fragmented adipose tissue has been promptly used and commercialized to offer minimally manipulated [35] options avoiding both cell expansion and enzymatic treatments. It is worth recalling, however, that optimal acquirement of the SVF is not always possible [26]. However, some authors have recently demonstrated [36,37,38,39,40,41,42] the efficacy of a medical device named Rigenera^®^ (CE certification, Class II, Human Brain Wave, Turin, Italy) which is able to obtain adipose micrografts enriched by cellular progenitors which are immediately available for common clinical practice. Adipose micrografts are characterized by high cell viability and are obtainable through mechanical disruption [25,43]. The aim of this work is to characterize the SVF obtained by a well-known commercial system (Rigenera^®^) through mechanical disruption of canine adipose tissue without substantial manipulations.

## 2. Materials and Methods

### 2.1. Isolation and Expansion of ASC

Adipose tissue samples (8 mL) were harvested from two different anatomical region (lumbar and thigh) of *n* = 6 dogs, donated by the owners and with appropriate informed consent to the University of Camerino, using a standard surgical procedure previously described [43]. The study was conducted according to the guidelines of the Declaration of Helsinki and approved by the Animal Welfare Organization (or OPBA) of Camerino University (protocol code 1D580.18A).

Each adipose sample was divided into two portions. The first portion was processed in the Rigenera^®^ technology: the device is composed of an engine in which the mechanical disaggregation is performed inside a disposable sterile capsule featuring steel blades rotating at 80 rpm and followed by filtration through 80 µm pores [25,43]. Specifically, 4 mL of lipoaspirate and 4 mL of complete culture medium Dulbecco Minimum Essential Medium (DMEM) (Sigma-Aldrich, Milan, Italy) containing 10% of Fetal Bovine Serum (FBS), 1% of a mix of penicillin/streptomycin 1:1 (GIBCO Life Technology, Monza, Italy) and 0.5% amphotericin B (GIBCO Life Technology, Monza, Italy) were added in the sterile capsule and Rigenera^®^ device was activated for 1 min. The obtained micrografts, were collected from the capsule by a syringe, filtered through a 70-µm nylon mesh, and centrifuged at 3000 rpm for 7 min. The supernatant was discarded, while cell pellet was resuspended in 1 mL of complete medium and then counted. The second portion of lipoaspirate was digested enzymatically as reported by Senesi et al. [26]. Briefly, 4 mL was digested with 1 mg/mL type I collagenase (GIBCO Life Technology, Monza, Italy) in Hank’s Balanced Salt Solution (HBSS) and 2% bovine serum albumin (BSA) at 37 °C for 45 min. The enzymatic action was neutralized adding complete medium. Then, the sample was centrifuged at 3000 rpm for 7 min, the supernatant was discarded, and the cell pellet was incubated with 3 mL of 160 mM NH4Cl at room temperature for 10 min to lyse the erythrocytes. After centrifugation, cells were resuspended in 1 mL of complete medium, filtered through a 70-µm nylon mesh, and counted. The obtained cells (Lumbar Rigenera, Thigh Rigenera, Lumbar Enzymatic Digestion (ED), Thigh ED) were plated on a 25 cm^2^ T-flask and incubated at 37 °C with 5% CO_2_. Three days after the cells’ extraction, the complete medium was changed and then every 48 h until 80% confluence and used for the subsequent analyzes.

### 2.2. Cells Yield

Cells obtained, both from the Rigenera^®^ and the ED process, were counted through the Trypan Blue exclusion method by dividing the number of viable cells per mL of processed fat. Data are expressed as number of viable cells/mL fat ± standard error of the mean (SEM).

### 2.3. Cell Colony Forming Unit Assay

Colony forming unit-fibroblast (CFU-F) assay was performed for tissue processed with Rigenera^®^ and ED. Briefly, isolated cells were plated into six-well culture plates at a density of 1000 cells/cm^2^ and cultured in the complete media. On the 15th day after plating, the total number of cell colonies (CFU-F, a cluster of at least 50 adhered and fibroblast-like cells) was rinsed with phosphate-buffered saline twice, fixed with 10% neutral buffered formalin for 30 min and then stained with toluidine blue (Sigma Aldrich, Milan, Italy) and counted. Colony forming efficiency (CFE) was calculated by dividing the number of colonies counted by the number of cells seeded × 100. Data are expressed as CFE ± SEM.

### 2.4. Proliferation Capacity

At day four from ASC isolation, cells were detached using Trypsin-EDTA 1% (GIBCO Life Technology, Monza, Italy) and replated at a density of 10,000 cell/cm^2^ plated into six-well culture (in triplicates). Cells were detached and counted with CytoSMART counter (Automated Image Based Cell Counter, version 1.5.0.16380, CytoSMART technologies B.V, Eindhoven, The Netherlands) after 24, 72, and 96 h. The population doubling time (pdt) was calculated using the following equation: pdt = [t (h) × log_2_]/log (N_f_/N_i_) (as reported in Martinello T. et al. 2010) [42], where N_i_ and N_f_ are initial and final cell numbers, respectively.

### 2.5. Immunophenotyping

After isolation, cells were counted and 2 × 10^5^ cells were placed in a tube for cytofluorimetric analysis. The pellet was washed with 1 mL of 1% FBS in PBS and then labelled with fluorescent-dyes conjugated antibodies in a final volume of 100 µL and incubated for 30 min in ice. This study examined specific antibodies: APC-conjugated CD90 (dilution 1:5), APC Alexa Fluo-conjugated CD73 (dilution 1:20), PE-conjugated CD34 (dilution 1:5), BV650-conjugated CD45 (dilution 1:20), and APC-conjugated MHC II (dilution 1:5). The antibodies were purchased from BD Biosciences, (Becton Dickinson Italy S.p.A., Milan, Italy). After the incubation, the pellet was rinsed, resuspended in 300 µL of 1% FBS in PBS, and transferred in flow cytometry tubes. The immunophenotyping was performed through a FACS canto II (Becton Dickinson Italy S.p.A., Milan, Italy).

### 2.6. Qualitative Analysis of Multipotency

The potential of the ASCs, obtained after Rigenera^®^ and ED, to differentiate into multilinear cell lineage (adipocytes, chondrocytes, and osteocytes) was evaluated by adding adipogenic, chondrogenic, and osteogenic media separately. ACSs were cultured until passage n.3 in order to remove peripheral blood contaminants and other non-adherent stromal cells, detached using Trypsin-EDTA 1% (GIBCO Life Technology, Monza, Italy), and replated in triplicates in multiwell plate with the above-mentioned different media.

Adipocyte differentiation was achieved after 16 days of culture of MSCs with adipogenic medium, containing 10^−6^ M dexamethasone, 10 µg/mL insulin, and 100 µg/mL 3-isobutyl-1-methylxanthine (Sigma Aldrich, Milan, Italy). Chondrocyte differentiation was achieved after 14 days of culture with the StemPro chondrogenesis differentiation kit (GIBCO Life Technology, Monza, Italy). Osteocyte differentiation was achieved after 21 days of culture with the StemPro osteogenesis differentiation kit (GIBCO Life Technology, Monza, Italy). The non-induced cells of the control group were cultured with the ASC complete medium (Dulbecco’s ModifiedEagle Medium (DMEM), 10% FBS, and 1% penicillin/streptomycin).

Oil Red O, Alcian blue, and Alizarin Red Stain were employed to identify adipocytes, chondrocytes, and osteocytes, respectively.

#### 2.6.1. Adipogenic Differentiation

5.000/cells were seeded on the circular glasses inside the six-well plate with complete media. After 24 h, the media was removed and adipogenic media was added and replaced every 24 h. To confirm adipogenic differentiation, after 14 days, the cells were fixed with 4% paraformaldehyde (PFA) for 30 min and washed, followed by staining with a solution of Oil Red O (Bioptica, Milan, Italy) for 30 min and hematoxylin (Bioptica, Milan, Italy) for 1 min. Cells were washed with buffer solution and fixed with aqueous mounting media. Images were obtained using optical microscopy (Olympus BX-51 microscope, equipped with a KY-F58 CCD camera, magnification 20×).

#### 2.6.2. Chondrogenic Differentiation

In this experiment, 1 × 10^6^ cells were seeded with 5 µL of complete media on the glasses inside the 24-well plate, after 2 h the chondrogenic media was added to the cells. To confirm chondrogenic differentiation, after 14 days, cells were fixed with 4% PFA for 30 min. After fixation, Alcian Blue 8GX (SigmaAldrich, Milan, Italy) was filtered and added to each culture well for 30 min, and the cells were washed with buffer solution followed by hematoxylin stain for 1 min. Alcian blue was used to stain the extracellular matrix glycosaminoglycan. The cells were then washed with normal water and fixed with aqueous mounting media. Images were obtained using Olympus BX-51 microscope, equipped with a KY-F58 CCD camera (Magnification 10×).

#### 2.6.3. Osteogenic Differentiation

5000/cells were seeded on the round glasses inside the 12-well plate with complete media. After 24 h, the media was replaced with an osteogenic medium followed by media change every 48 h. To confirm osteogenic differentiation, after 21 days, cells were fixed with 4% PFA for 30 min and incubated in 0.2% Alizarin Red S (SigmaAldrich, Milan, Italy) for 15 min and hematoxylin for 1 min Then, they were washed with PBS (GIBCO Life Technology, Monza, Italy), and fixed with aqueous mountant. Images were obtained using optical microscopy (Olympus BX-51 microscope, equipped with a KY-F58 CCD camera, magnification 4×).

### 2.7. Real-Time PCR (Genes Involved in Inflammation or Anti-Inflammation)

Total RNA was extracted form Rigenera and ED obtained cells using Total RNA Purification Plus Kit (Norgen Biotek Corporation, Thorold, ON, Canada). The cDNA was synthesized starting from 500 ng of total RNA with SensiFASTTM cDNA Synthesis kit (Bioline GmbH, Luckenwalde, Germany) using LifePro Thermal Cycler (Bioer Technology, Hangzhou, China). Real-time PCR of genes involved in inflammation or anti-inflammation was performed. Canine primers were selected for each target gene with Prime 3 software. Canine primers selected were as follows (Table 1).

Thermal cycling conditions: denaturation at 95 °C for 2 min; 40 cycles of denaturation at 95 °C for 5 s; annealing at 60 °C for 10 s; and elongation at 72 °C for 20 s. Data analysis was performed using the ΔΔCt method using transferrin receptor (TFRC) as internal reference. Results were reported as fold regulation of target genes in the test group (product) compared with the control group (canine fibroblasts in monolayer culture).

### 2.8. Statistical Analysis

All data were analyzed using a one-way ANOVA test and showed a *p*-value < 0.05, capable of confirming their statistical significance. Repeatability was represented as a standard deviation to calculate differences between measurements using SPSS 16.0 software (SPSS Inc., Chicago, IL, USA) for assessment.

## 3. Results

### 3.1. Cellular Yield, CFE and Proliferation Capacity

**Figure 1:**
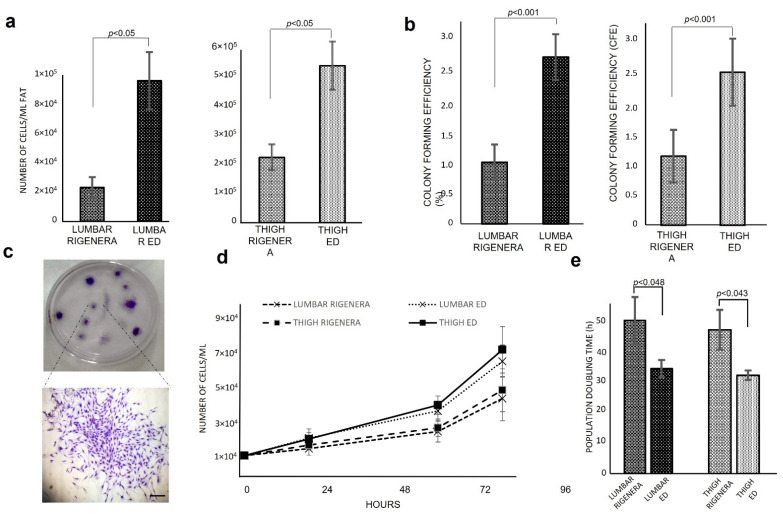
Cells yield, CFE and proliferation capacity of Rigenera^®^ obtained from lumbar and thigh region. (**a**) Cell yield of Rigenera^®^ and ED product. (**b**) CFE calculated based on CFU-F assay. (**c**) Representative petri dish of CFU-F assay and an amplified image of a CFU-F observed with light microscope stained with Toluidine Blue (4× magnification). (**d**) Proliferation capacity after 24, 72, and 96 h and (**e**) population doubling time analysis. Data are expressed as average ± SEM.

The cell yield of freshy isolated micrografts from Rigenera^®^ device was 2.23 × 10^4^ ± 6.94 × 10^3^ cells/mL Fat 2.29 × 10^5^ ± 4.54 × 10^4^ cells/mL Fat (Figure 1a), for lumbar and thigh, respectively. Compared to ED the cell yield of Rigenera^®^ results were 23.9 ± 3.2% and 41.6 ± 4.5% for lumbar and thigh, respectively. Moreover, the cell yield obtained from the thigh region was 10 and 6 times higher than the lumbar region for Rigenera^®^ and ED, respectively. To analyze the clonogenic potential of Rigenera^®^ cells, CFU-F assay was performed and CFE was evaluated (Figure 1b). The ED method allowed cell isolation with 2.58 ± 0.55% and 2.77 ± 0.47% of CFE, for lumbar and thigh, respectively, while Rigenera^®^ cells presented a clonogenic efficiency of 1.17 ± 0.44% for lumbar region and 1.2 ± 0.29% for the thigh. The difference between lumbar and thigh region was not statistically significant. Figure 1c represents a clone for CFU-F assay. To better characterize the cellular products, the proliferation capacity and the time required by cells to duplicate in number were estimated. As shown in Figure 1d, ED cells required less time to duplicate when compared to Rigenera^®^ cells. As reported in Figure 1, the population doubling time mean value was 50.17 ± 7.8 h and 47.02 ± 6.4 h for Rigenera^®^ lumbar and thigh, respectively. ED cells have a population doubling time 1.4 times higher compared to Rigenera^®^ (34.46 ± 4.8 and 32.16 ± 2.58 h for ED lumbar and thigh, respectively). Statistically significant differences between the lumbar and thigh were not detected.

### 3.2. Immunophenotyping

**Figure 2:**
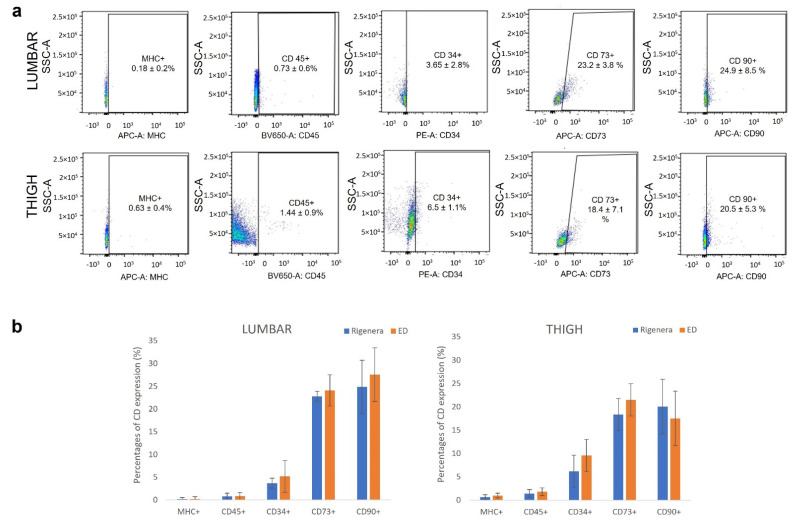
Immunophenotype analysis. (**a**) Representative set of dot-plot from one individual from lumbar and thigh region of Rigenera^®^ product. Results show that cultures were negative for MHC class II, less positive for CD45 and CD34, and positive for CD73 and CD90. (**b**) Percentage of positive cells to CD markers (as average of the samples) after Rigenera^®^ and ED treatment. Results are expressed as average ± SEM of *n* = 6 samples; no significant statistical differences in the marker expression level were found between Rigenera^®^ and ED treatment.

The relative expression percentages of surface markers of Rigenera^®^ micrografts analyzed by flow cytometry are shown in Figure 2a. The presence of surface molecules was analyzed using specific monoclonal antibodies against MHC II, CD45, CD34, CD73, and CD90. In the cell population examined, MHC II was not expressed and CD45 was poorly expressed, while the hematopoietic marker CD34 (endothelial cells, pericytes and potential ASCs) was expressed, especially in cells extracted from thigh region (6.5 ± 1.1%). Cells were positive for the mesenchymal stem cells marker CD 73 (23.2 ± 3.8% and 18.4 ± 7.1 for lumbar and thigh, respectively) and CD 90 (24.9 ± 8.5% and 20.5 ± 5.3 for lumbar and thigh, respectively) (Figure 2a). Comparing the surface marker expression profiles of cells obtained after Rigenera^®^ and ED, no significant statistical differences were found (Figure 2b).

### 3.3. Qualitative Analysis of Multipotency

**Figure 3:**
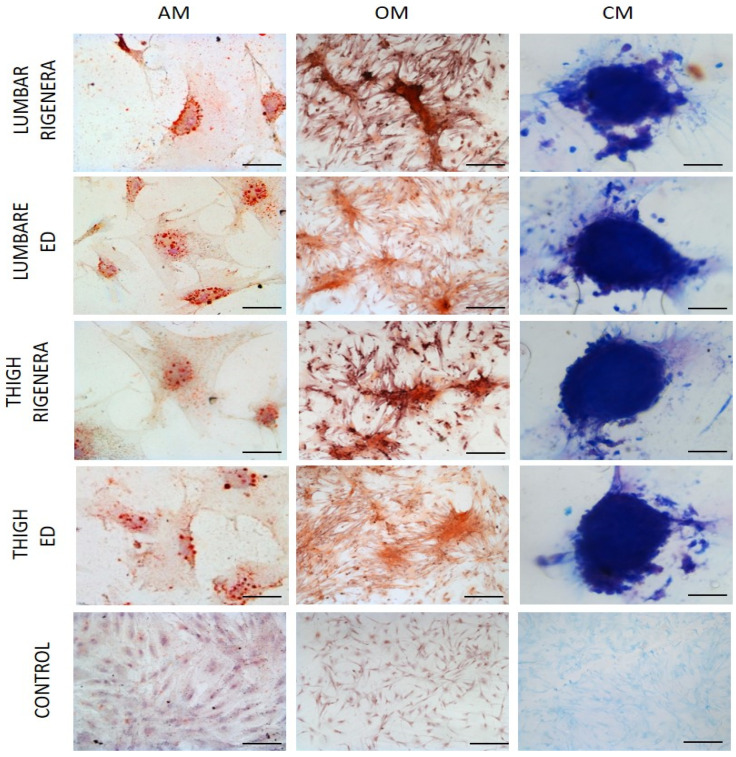
Representative photographs of differentiation capacity of ASCs obtained with Rigenera device. AM, adipogenic medium; CM, chondrogenic medium; OM, osteogenic medium. Control cultures were maintained in normal medium. All of the samples were stained with Oil Red O, Alzarin Red and Alcian Blue for adipo-, osteo- and chondrogenic differentiation, respectively. Scale bar: AM, 5 µm; OM, 20 µm; CM, 50 µm; Control, 20 µm.

To evaluate the multi-potency of Rigenera^®^ micrografts, ASCs were exposed to adipogenic, osteogenic, and chondrogenic medium. As shown in Figure 3 all samples were able to differentiate to mesodermal lineages. Oil Red O staining confirm the adipogenic differentiation and red lipid droplets were clearly visible in the cytoplasm of cells; alizarin red staining highlighted the extracellular matrix calcification typical of osteogenic differentiation, while chondrogenesis was observed by deposing sulfated proteoglycan-rich matrix stained with alcian blue. No significant differences were qualitatively observed between Rigenera^®^ and ED and between the lumbar and thigh region.

### 3.4. Real-Time PCR (Genes Involved in Inflammation or Anti-Inflammation)

**Figure 4:**
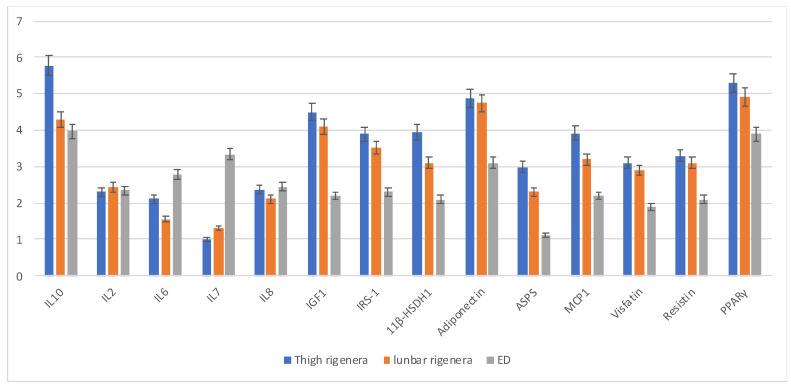
Anti-inflammatory properties of Rigenera^®^ and ED products. Representative results showed that both products generate more anti-inflammatory cytokines such al IL10 compared to the other anti-inflammatory ones (IL2-8-7-8). Specifically, Rigenera -acquired micrografts yield a lower production of inflammatory marker IL7 and a higher quantity of anti-inflammatory IL10. (Results are expressed as average ± SEM of *n* = 3 samples).

The positive mechanisms of MSC to contrast the inflammation were evaluated through gene expression of the anti-inflammatory (IL10) and inflammatory (IL2-6-7-8) cytokines at the moment of substance injection. Results reported in Figure 4 show that both technologies gave rise to products that revealed greater anti-inflammatory activity (IL10) compared to the inflammatory IL (2, 6, 7, 8), confirming that MSCs act as an effective biological stimulating their paracrine activity. In the same way, 11β-HSD1, PPARγ, and adiponectin are very expressed, as they are known to be present in adipose tissue but also due to the fact that they are related to modulate hMSC metabolism to enhance their immunomodulation and therapeutic efficacy. MCP1 activates macrophages therefore it is involved in anti-inflammatory activity, in particular the expression of MCP1, associated with expression of anti-inflammatory chemokines such as Visfatin and Resistin, can modulate the plasticity of macrophages that depend on various microenvironmental signals. Moreover, IGF1 can modulate cartilage and subchondral bone during the regeneration processes of cartilage defects, especially in the early stages of the disease, and its function is related to its receptor IRS-1. The expression of ASPS, which is normally linked to primordial bone differentiation, demonstrates that the cells extracted by the Rigenera method are of a mesenchymal nature. In conclusion, the comparison between the two techniques demonstrated that Rigenera Technology showed a higher expression of IL10 (anti-inflammatory) compared to the ED generated and a lower expression of IL7, but also a higher expression of immunomodulatory factors.

## 4. Discussion

Regenerative medicine involves a number of activities towards repair, regeneration, or substitution of damaged tissue or organs with the use of ex-vivo manipulated cells [44].

Our study objective is to investigate a well-known commercial system (Rigenera^®^) to obtain SVF by mechanical disruption of canine adipose tissue without substantial manipulations comparing, in addition to the obtained micrografts to ASCs.

Before the procedure, blood and any cellular debris are removed to depurate the adipose tissue which will then prevent inflammatory activity and maintain graft sustainability via various enzymatic and mechanical techniques. Typically, collagenase enzymatic digestion is the most common and effective method available. However, it is not without limitations.

The enzymatic digestion via collagenase is able to eradicate the stem-cell niche competent in relating to the surrounding cell environment and enhancing cell viability, expansion, and differentiation [45]. Furthermore, GMP guidelines of the European Parliament and Council (EC Regulation no. 1934/2007) strictly indicate use of techniques with minimal cell manipulation within a clinical context, thus excluding the adoption of enzymatic methods.

Accordingly, numerous efforts have been made to develop medical devices able to mechanically disaggregate a tissue. The Rigenera^®^ (HBW, Turin, Italy) system presents a disposable, motor-driven sterile device that deserves to be taken into account [46]. Injectable micrografts are instantly produced and consist of fragments of adipose tissue with a dimension of 80 µm capable of stimulating the regeneration of damaged tissue [46].

This study thus focused on the in vitro characterization and comparison between the proposed treatment and the gold standard enzymatic digestion in terms of cell yield, CFE, proliferation capacity, immunophenotyping, multipotency and anti-inflammatory activity. Additionally, adipose tissue samples were harvested from two different anatomical regions (the lumbar and the thigh) to evaluate differences in the regenerative potential.

Our results showed that the cell yield, and thus the number of CFU, the proliferation capacity, and the time required by cells to duplicate, were higher for ED cells compared to Rigenera^®^ cells, pointing out the main limitation of the treatment. Nevertheless, this restriction was not due to the Rigenera^®^ technology alone, but as reported in literature [22,23], all non-enzymatic methods show lower cell recovery compared to enzymatic methods.

Some authors [23,47] clarified that the differences may be partly related to the SVF cell site within adipose tissue in the perivascular niche. Similar cell release from the perivascular niche is not commonly observed within the mechanical procedures of SVF isolation, since the enzymatic method does not allow for complete disruption of the extracellular matrix due to the use of proteolytic enzymes digesting the extracellular matrix and withal consolidating the adipose tissue. In the light of these findings, the setting and application of an appropriate protocol is imperative.

Besides, in terms of cell yield, a slight difference between cells obtained from the lumbar and the thigh regions was detected from both ED and Rigenera^®^ in favor of the thigh part, most likely attributable to the thigh being a pristine reserve site regarding extraction, and moreover to the uniformity of the adipose tissue and a thinner collagen matrix, consequently yielding more accessible tissue [41].

Herein, we confirm how cells extracted via the Rigenera^®^ device may easily differentiate. Results showed the comparable ability of both ED and Rigenera^®^ cells to differentiate to mesodermal lineages, through staining with oil red o, alzatin red and alcian blue for osteo-adipo and chondrogenic recognition, respectively. For this reason, multipotent cells will efficiently regenerate and repair tissue.

Moreover, the immunophenotypic analysis attested that cells from Rigenera^®^ were highly positive for the mesenchymal stem cells markers CD73 and CD90, low positive for hematopoietic CD45 and CD34, and negative for MHC class II antibodies, playing a role in immune responses.

Finally, the main strength of the Rigenera^®^ technology relies on a 35% higher expression of the IL10 gene, with anti-inflammatory activities compared to the ED protocol. This evidence suggests a potential improved clinical therapy capable of modulating inflammation and immune responses. Interleukin-10, also associated with the expression of ASPS, IGF1 and its receptor IRS-1, clearly has chondroprotection activity, stimulating the expression of collagen type II and proteoglycan and regulating the maintenance of tissue integrity [48]. Moreover, our results demonstrated that adipose tissue also hosts chemokines such as 11β-HSD1, PPARγ, and adiponectin which have been shown to contribute to immunoregulation through modulation of hMSC metabolism [49,50], but also chemokines such as MCP1, Visfatin, and Resistin which have been shown to contribute to immunoregulation through modulation of macrophages plasticity [51]. Beyond that, adiponectin, ASPS, MCP1, Visfatin, and Resistin, when taken together, prove that they can contribute to the diagnosis of OA, which is consistent with our results. Furthermore, the levels of OA can also be directly related to the degree of OA [52], and therefore in the future they can be used as biomarkers to assess the severity of OA.

Finally, except for the cell yield as mentioned above, the study did not show statistically significant differences between the lumbar and the tight regions from which samples were collected.

## 5. Conclusions

Autologous, adipose tissue derived micrografts, obtained with the Rigenera^®^ technology, represent an innovative approach that introduces a completely novel concept in regenerative medicine, showing the safety and potential benefits of minimal tissue manipulation. The impressive in vitro outcomes demonstrated that this particular technology may be used to restore functionality and relieve pain in dogs with severe OA. Additionally, such a procedure is a straightforward, rapid, and sustainable one-step method (as well as being a minimally invasive and secure option) compared to the enzymatic method which, albeit consolidated as a method for 40 years, remains inoperable in clinical settings due to the time-consuming applications, legal limitations, and scientific constraints. Our future work will involve an in -vivo experimental study aimed at performing clinical, eco-graphic, and sonographic evaluations in the dog model.

## Figures and Tables

**Table 1:** Selected canine primers.

Gene	Sequence FOR (5′–3′)	Sequence REV (5′–3′)	Length (bp)
IL10	CCGTTGCGCAGGCAGTGTG	TGTCTAACTTGTAGATCCTGACC	206
IL2	CATTGCCCACTCCTCTCTGAA	GTTTCTTTCTCTTCCTCACTGACCA	167
IL6	GCCTTGGAAACGCAAACTCG	GTCCCTGTATGTCCTCCCTTC	219
IL7	CCATCCTATTCTAGACCGTTGAGAG	GCCACCATAAGAACATTTGCATCA	211
IL8	TCTCCTGCTCGCCTTCTTC	CCTAAGTAATCGAGTTCCGTGCTG	147

## Data Availability

The clinical data used to support the findings of this study are included within the article.

## Abbreviations

OA	Osteoarthrosis
PRP	Platelet Rich Plasma
MSC	Mesenchimal Stem Cells
ASC	Adipose derived Stem Cells
SVF	Stromal Vascular Fraction
ECM	Extra-Cellular Matrix
GMP	Good Manufacturing Practice
ED	Enzymatic Digestion
CFU-F	Colony Forming Unit-fibroblast
CFE	Colony Forming Efficiency
TFRC	Transferrin Receptor
IGF1	Insulin-like growth factor
IRS-1	Insulin receptor substrate-1
11β-HSDH1	11beta-hydroxysteroid dehydrogenase type 1
ASPS	Acylation stimulating proteins
MCP1	Monocyte chemoattractant Protein 1
PPARγ	Peroxisome proliferator-activated receptor gamma
