# Correction: De Francesco et al. In Vitro Characterization of Canine Microfragmented Adipose Tissue Non-Enzymatically Extracted from the Thigh and Lumbar Regions. *Animals* 2021, *11*, 3231

**DOI:** 10.3390/ani12060673

**Published:** 2022-03-08

**Authors:** Francesco De Francesco, Valentina Riccio, Reetuparna Biswas, Alice Busato, Caterina Di Bella, Evelina Serri, Andrea Sbarbati, Barbara Zavan, Michele Riccio, Angela Palumbo Piccionello

**Affiliations:** 1 Hand Surgery Unit, Department of Plastic and Reconstructive Surgery, Azienda ‘Ospedali Riuniti di Ancona’, 60126 Ancona, Italy; michele.riccio@ospedaliriuniti.marche.it; 2 School of Biosciences and Veterinary Medicine, University of Camerino, 62032 Matelica, Italy; valy.riccio91@gmail.com (V.R.); caterina.dibella@unicam.it (C.D.B.); evelina.serri@unicam.it (E.S.); angela.palumbo@unicam.it (A.P.P.); 3 Department of Neuroscience, Biomedicine and Movement, Human Anatomy and Histology Section, University of Verona, 37179 Verona, Italy; reetuparna.biswas@univr.it (R.B.); alice.busato@univr.it (A.B.); andrea.sbarbati@univr.it (A.S.); 4 Department of Morphology, Surgery and Experimental Medicine, University of Ferrara, 44121 Ferrara, Italy; barbara.zavan@unife.it

## Addition of an Author

**Reetuparna Biswas** was not included as an author in the original publication [1]. The corrected Author Contributions Statement appears here. The authors apologize for any inconvenience caused and state that the scientific conclusions are unaffected. The original publication has also been updated.
